# Obinutuzumab, High-Dose Methylprednisolone (HDMP), and Lenalidomide for the Treatment of Patients with Richter’s Syndrome

**DOI:** 10.3390/cancers14246035

**Published:** 2022-12-08

**Authors:** Benjamin M. Heyman, Michael Y. Choi, Thomas J. Kipps

**Affiliations:** 1 Division of Regenerative Medicine, Department of Medicine, UC San Diego Moores Cancer Center, UC San Diego, La Jolla, CA 92093, USA; 2 Division of Hematology/Oncology, Department of Medicine, UC San Diego Moores Cancer Center, UC San Diego, La Jolla, CA 92093, USA; 3 Division of Hematology/Oncology, Center for Novel Therapeutics, UC San Diego Moores Cancer Center, UC San Diego, La Jolla, CA 92093, USA

**Keywords:** CLL, Richter’s Syndrome, chemotherapy-free

## Abstract

**Simple Summary:**

Patients with chronic lymphocytic leukemia who develop Richter’s Syndrome have a poor prognosis. Traditional chemo-immunotherapy approaches yield poor response rates and increase toxicity. Novel, non-myelosuppressive regimens are needed to improve outcomes and reduce toxicity. Here, we present our experience administering the novel chemotherapy-free combination of obinutuzumab, high-dose methylprednisolone, and lenalidomide for patients with Richter’s Syndrome.

**Abstract:**

**Background:** For patients with Richter’s Syndrome (RS), a durable response is rarely achieved with standard therapies. Significant efforts have focused on the development of novel treatments with reduced toxicity. We describe our experience using the novel combination of obinutuzumab, high-dose methylprednisolone (HDMP) and lenalidomide (len) in patients with RS. **Patients and Methods:** Eligible patients included adults with biopsy-proven RS. Patients received obinutuzumab 1000 mg × 8 doses. All patients received HDMP 1000 mg/m^2^ on days 1–5 of cycles 1–4. Patients were administered len PO daily, starting at a dose of 5 mg. Starting on C2D1, the dose increased every 2 weeks in 5 mg increments to a maximum of 25 mg PO daily. **Results**: Seven patients were treated. The median dose of len was 10 mg and the median number of cycles of treatment completed was 2. The most common grade 3/4 adverse events were neutropenia (29%) and pulmonary embolism (29%). The overall response rate for the entire cohort was 43% (95% CI, 10–82%). All patients who achieved a response underwent consolidative autologous or allogeneic stem cell transplant and remain in remission to date. **Conclusions**: The combination of obinutuzumab, HDMP, and len is a well-tolerated, outpatient regimen that could serve as a bridge to transplantation, or as palliation for transplant-ineligible patients with RS.

## 1. Introduction

The treatment for patients with chronic lymphocytic leukemia has undergone a dramatic shift over the past two decades with the emergence of therapies that target B-cell receptor (BCR) signaling pathways [1]. Specifically, Bruton tyrosine kinase inhibitors (BTKi) as well B-cell lymphoma-2 inhibitors (BCL-2i) have led to dramatic increases in patient survival, as well as improved tolerability of treatment [1]. These novel therapies have also help reduced disease-related complications of immunodeficiency and infection [2]. However, despite improvements in CLL-directed therapies and survival, neither the administration of BTKi or BCL-2i appear to mitigate the risk of histological transformation to aggressive lymphoma (Richter’s Syndrome) [3,4,5]. Richter’s Syndrome (RS) represents a transformation for CLL, typically into diffuse large B-cell lymphoma (DLBCL) or, rarely, into classic Hodgkin lymphoma (cHL) or plasmablastic lymphoma [6]. Historically, in the era of chemo-immunotherapy, up to 20% of previously treated CLL patients will undergo RS during their disease, with an incidence of 0.5% in untreated patients per year and 1% per year in treated patients. The median overall survival (OS) is dismal, ranging from 3–11 months, even with traditional chemo-immunotherapy approaches [7]. In the era of novel agents, the reported incidence of RS has not significantly changed, varying between 3% to 20%, irrespective of treatment modality (BCL2i vs. BTKi vs. combination therapy) [3,4,5] It does appear that RS may occur earlier when patients are receiving kinase inhibitors, and less often when administered as front-line treatment versus in the relapsed/refractory (R/R) setting [8]. 

The molecular mechanism that causes RS to occur in CLL are not clearly understood, but the vast majority (≥80%) are clonally related [9]. Furthermore, molecular biomarkers demonstrate that RS DLBCL frequently harbor high-risk mutations in TP53 (~60%), NOTCH1 (~30%), and MYC (~50%) [10]. These mutations contribute to treatment resistance. Standard R-CHOP chemo-immunotherapy as a first-line therapy for RT has demonstrated response rates of 50% to 60%, however these responses are short, with poor OS ranging only from 15 to 21 months [7]. Intensification to R-EPOCH, R-DHAP or R-hyper-CVAD has not resulted in improved OS, but with increased toxicity [11,12,13]. Consolidation approaches with either autologous or allogeneic stem cell transplantation have demonstrated improved 3-year survival rates [14]. However, these studies are limited because most patients with RS will not achieve a good enough response in order to undergo consolidation transplant. Furthermore, as CLL is a disease of the elderly, many patients will either be too old or have significant co-morbidities by the time they are diagnosed with RS such that they will not be eligible to undergo consolidative transplant treatment strategies [15]. Thus, treatment strategies that improve responses, and with a favorable toxicity profile, are urgently required for this high-risk patient population. We previously reported a case series of two patients with RS who were successfully treated with the novel chemotherapy free-regimen of obinutuzumab, high-dose methylprednisolone (HDMP), and lenalidomide (len), achieving complete remissions (CRs) and subsequently undergoing allogeneic stem cell transplant [16]. Given the exciting preliminary results, we now go on to report the combined follow-up results of the initial case series and subsequent phase 1 clinical trial of the novel combination of obinutuzumab, HDMP, and len for the treatment of patients with RS.

## 2. Materials and Methods

### 2.1. Eligibility Criteria

Three patients with RS were initially treated as a case series with a combination of obinutuzumab, HDMP, and len. After the results demonstrated preliminary efficacy, a phase 1 open-label and non-randomized clinical trial was developed. To be eligible to enroll in the study, subjects had to 18 years or older, with an Eastern Cooperative Oncology Group (ECOG) performance status between 0–2. Subjects were required to have a histological diagnosis of RS to be enrolled. Prior treatment specifically for RS was not required, and subjects could have had prior treatment for either CLL, RS or both. Subjects had to have had measurable disease as defined as an FDG-avid lesion, lymph node greater than 1.5 cm in greatest diameter, or clonal large B-cells in peripheral blood or bone marrow. Patients with positive Hepatitis B and C serology were allowed in the study if they had negative viral PCR testing. Subjects had to have adequate organ function to enroll in the study as defined as follows:

Total bilirubin < 3 × the upper limit of normal (ULN);

AST or ALT ≤ 2.5 × ULN; and

Creatinine clearance > 30 mL/min as calculated using the Cockcroft-Gault Formula.

Adequate baseline bone marrow function: Platelets ≥ 30,000 cells/mm^3^; Absolute neutrophil count > 750 cells/ uL, unless due to infiltration of bone marrow by CLL or RS cells. Growth factors or transfusions were permitted to meet treatment parameters.

Lastly, as subjects would be administered len there were specific len-related inclusion criteria. Subjects were required to take anti-thrombotic prophylaxis with either aspirin, warfarin, low-molecular weight heparin, or equivalent. All study patients had to register to participate in the REVLIMID REMS^®^ program, and be willing and able to comply with the requirements of REMS, including pregnancy testing and contraceptive use.

The study was approved by the institutional review board at the University of California San Diego (HRPP#161265). Each patient provided written informed consent. The study was conducted in accordance with the recommendations of Good Clinical Practice and the Declaration of Helsinki. The study was registered at ClinicalTrials.gov (accessed on 28 November 2022) (NCT03113695).

### 2.2. Study Design

This was a phase 1 open-label and non-randomized clinical trial. The primary objective of the study was to determine the safety and tolerability of the combination of obinutuzumab, HDMP, and len in patients with RS. The primary endpoint of the study was the rate of dose-limiting toxicities (DLTs) from the combination. All patients were assigned to the same dose, including a maximum len dose of 25 mg PO daily. There was no inter-patient len-dose escalation, but there was an intra-patient dose ramp-up, starting at a 5 mg dose. Previous studies in CLL have shown that not all patients tolerate or require the full 25 mg dose [17]. Therefore, the maximum tolerated dose of len in this regimen was not the primary endpoint of the study. Secondary endpoints included the overall response rate (ORR), progression-free survival (PFS), and OS at 12 months and 24 months after the start of treatment. Other secondary endpoints included the percentage of patients that were able to receive a subsequent stem cell transplant. 

All patients received obinutuzumab 1000 mg × 8 doses (for cycle 1, the first dose is split on day 1 as 100 mg and on day 2 as 900 mg; then 1000 mg on days 8 and 15). On subsequent cycles, 1000 mg of obinutuzumab was administered on day 1 of each cycle. All patients received HDMP 1000 mg/m^2^ on days 1–5 of cycles 1–4. Patients were administered len PO daily. The starting dose of len was 5 mg PO daily. Starting on C2D1, the dose increased every 2 weeks in 5 mg increments to a maximum of 25 mg PO daily in the absence of ongoing grade 3 or higher neutropenia or thrombocytopenia. Patients continued len until disease progression, unacceptable toxicity, or subsequent therapy (including stem cell transplant). All cycles were 28 days. Per protocol, patients could stop study treatment to receive consolidation stem cell transplant prior to completing 6 cycles of treatment (Table 1).

Adverse events (AEs) were monitored throughout therapy, but dose-limiting toxicity (DLT) determination was based on events during cycles 1 through 3. A DLT was defined as a grade 3 nonhematologic toxicity, or any grade ≥ 3 hematologic toxicities lasting longer than 7 days except for lymphopenia. Reversible grade 3 infusion reactions were also not considered DLTs. Non-hematologic adverse events were evaluated based on CTCAE4 criteria. Hematologic adverse events were evaluated based on iwCLL criteria [18]. As this was a hypothesis-testing study for safety, no formal sample size or power calculations were made, and the sample size was not based on power or Type I error levels. A sample size of 10 patients was selected as a preliminary assessment of safety and antitumor activity. If 0 to 1 DLTs were observed in the first 3 subjects, 3 additional subjects would be enrolled. If 0 to 1 DLTs were observed in the first 6 subjects, we would proceed to phase II of the study. If ≥2 DLTs were observed, the study would be stopped. 

### 2.3. Patient Evaluation 

All patients who received any amount of obinutuzumab, HDMP, and len were included in the response analysis. Response and progression were evaluated by Lugano response criteria for non-Hodgkin’s Lymphoma [19]. Additionally, for patients with concurrent measurable CLL, response assessment was also assessed by iwCLL criteria [18]. Restaging laboratory, bone marrow biopsies, and radiographic studies were assessed 28 days following completion of treatment. 

## 3. Results

### 3.1. Patient Characteristics

A total of seven patients were treated (three off-label and four on the phase 1 clinical trial). Overall, the median age of the patients who were treated was 68 years (range, 54–74). For six of the patients the histological subtype of transformation was DLBCL, while for one patient it was cHL. The median Rai stage was two (range, 1–4). Most patients had high-risk cytogenetics, including: Trisomy 12 in 43% of patients, del17p in 43% of patients, complex cytogenetic abnormalities in 57% of patients, and del11q in 57% of patients. Unmutated IGHV was identified in 29% of patients. The median time to transformation was 66 months (range, 0–423). The median number of prior CLL treatments was 1 (range, 0–8). Three patients were previously treated with a novel kinase inhibitor. The median CLL-IPI score was 4 (range, 4–10) At the time of transformation, 71% of the patients had advanced stage disease from their RS. Zero patients had double/triple HIT lymphoma, and one patient had a double expresser lymphoma. All patients were found to be negative for Epstein Barr-Virus at the time of RS. The median number of previous RS treatments was zero, (range, 0–2). Most patients had either intermediate or high-risk disease by Richter’s Prognostic score (intermediate = 3, high-risk = 2) [20] (Table 2). 

The median number of cycles administered was two (range, 1–5). Three patients completed at least four cycles of treatment. The median dose of len was 10 mg (range, 5–20 mg). Reasons for treatment cessation prior to completion of all planned 6 cycles included: two (29%) patients for disease progression, three (43%) patients to proceed for consolidative stem cell transplant, and two (29%) patients who had stable disease but changed therapy as decided by the treating physician. With a median follow-up of 92 months, the median PFS was 5 months (range, 1–6.5); and the median OS was 17 months (range, 2.4–104.9) (Table 3).

### 3.2. AEs

Of the four patients enrolled in the clinical trial, only one patient had a DLT with grade 3 pulmonary embolism; this was incidentally found despite appropriate anti-thrombotic prophylaxis. Another patient also developed a pulmonary embolism in the setting of grade 3 thrombocytopenia and grade 3 gastrointestinal hemorrhage. This did not allow for the use of anti-thrombotic prophylaxis and, despite severe thrombocytopenia, ultimately developed a pulmonary embolism. The same patient also developed grade 3 infectious complications in the setting of neutropenia. Grade 3/4 hematologic AEs were infrequent, with 29% developing grade 3/4 neutropenia and only 14% developing grade 3/4 anemia and thrombocytopenia. Interestingly, rates of grade 1/2 hematological toxicity were also low, with only 29% of patients developing grade 1/2 neutropenia, anemia, or thrombocytopenia. The most common AE was fatigue, which occurred in 57% of patients. Infectious complications occurred in 29% of patients. Overall, there was a low incidence of grade 3/4 AEs (Table 4).

### 3.3. Efficacy

All patients were evaluated for efficacy. The ORR for the entire cohort was 43% (95% CI, 10–82%). Two patients achieved a CR; one with DLBCL and one with cHL. One patient had a partial response (PR). Two patients achieved stable disease (SD), and two patients had overt disease progression (Table 3). Of the three patients who achieved a response, all three went on to receive consolidative stem cell transplant. Two received allogeneic stem cell transplants and one received an autologous stem cell transplant. Both patients who underwent allogeneic stem cell transplant received a conditioning regimen of fludarabine, melphalan and anti-thymocyte globulin followed by a matched unrelated-donor transplant. The one patient who underwent an autologous stem cell transplant received carmustine, etoposide, cytarabine, and melphalan (BEAM) conditioning. The patient who underwent autologous stem cell collection after completion of len-based treatment for RS had successful stem cell mobilization and collection prior to transplant. The dose of len was not predictive of response in the three patients who did respond, with responses seen at doses of 5 mg, 15 mg, and 20 mg. Interestingly, one of the patients who was removed from the treatment at the physician’s discretion, remained on len at a dose of 10 mg daily and had pembrolizumab added to their treatment regimen, achieving a partial remission that was durable for >1 year. At the time of the last follow-up, all three patients that had undergone either autologous or allogeneic stem cell transplant are currently alive and disease-free.

## 4. Discussion

We report the combined results of a three-patient case series and a phase 1 clinical trial of the treatment combination of obinutuzumab, HDMP, and len for patients with RS. We chose to investigate this combination for patients with RS for several important reasons. One of the main challenges for patients with RS is that it frequently arises in the background of systemic CLL. Thus, effective treatment for RS requires treatment for the aggressive lymphoma while attempting to treat the underlying CLL or, at a minimum, not exacerbate complications related to CLL. Furthermore, patients with CLL have limited myeloid reserves, and are at increased risk of infections [18]. Moreover, patients with RS frequency harbor TP53 mutations, which contribute to chemo-immunotherapy resistance. Thus, traditional approaches to RS that have included R-CHOP-based chemo-immunotherapy are limited as they only exacerbate immunodeficiency, and do not adequately treat the underlying disease [7]. We have previously demonstrated the addition of HDMP to an anti-CD20 monoclonal antibody is an effective non-myelosuppressive treatment combination for patients with CLL [21,22]. Len is an active agent in both DLBCL and CLL [23,24]. Preclinical research has demonstrated that len impairs proliferation of malignant B-cells via a cereblon/p21-dependent mechanism independent of a functional p53.1 [25]. Both HDMP and obinutuzumab also target malignant B-cells through a p53-independent manner. This provided rationale for our approach to combine obinutuzumab, HDMP, and len for patients with RS as a means to effectively treat both RS and CLL, while minimizing myeloid toxicity and infectious risks.

The results of our report demonstrate that the regimen was well-tolerated and found to have clinical activity for patients with RS. No new safety signals were observed with the regimen; however, two patients were diagnosed with pulmonary embolism. This is an important potential side effect of treatment with len-based regimens, and while aspirin was administered for most patients as anti-thrombotic prophylaxis, if the regimen is used in the future, anti-thrombotic prophylaxis with a direct oral anticoagulant or enoxaparin may be more appropriate. Overall, the incidence of hematological toxicity and infectious complications was lower compared to traditional chemo-immunotherapy approaches for patients with RS. The incidence of grade 3/4 neutropenia was 29%, with only one patient developing febrile neutropenia. By comparison, the incidence of grade 3/4 neutropenia in elderly patients treated with R-CHOP is as high as 50%, with approximately 30% of patients developing grade 3/4 infectious complications and 50% requiring IV antibiotic administration. For patients treated with the more intensive regimens R-EPOCH, R-DHAP or R-hyper-CVAD, the rates of hematologic and infectious complications are even higher [7,12,13]. Comparing the safety of the obinutuzumab, HDMP, and len combination to other len-based regimens demonstrates a similar toxicity profile. Len plus rituximab has been investigated in patients with CLL, with approximately 48% (72% grade 3/4) developing neutropenia and 28% developing infectious complications. The incidence of anemia was 24% (21% grade 3/4) and that of thrombocytopenia 16% (16.7% grade 3/4) [23]. Recently, the L-MIND study investigated len in combination with the anti-CD19 monoclonal antibody tafasitamab for patients with R/R DLBCL; again, thew hematologic and infectious side-effect profile seen was similar to that for the combination of obinutuzumab, HDMP, and len [26].

Our study added HDMP to the backbone of obinutuzumab and len, a doublet that has demonstrated clinical efficacy in patients with aggressive B-cell lymphomas. Although the number of patients treated with our regimen is small, the clinical activity was encouraging, with 3 patients achieving a response (ORR 43%), and two patients achieving SD (SD 29%). All 3 patients who achieved a response were able to proceed to either allogeneic or autologous stem cell transplant and remain in remission to date. This compares favorably to reported len-based regimens for patients with aggressive B-cell lymphoma. Single-agent len was initially studied in patients with RS, and found to have an ORR of 0% [27]. Len and rituximab (R2) in patients with R/R DLBCL has demonstrated ORR of approximately 30–35%, with a median PFS of approximately 3.7 months [24]. In the L-MIND study, len plus tafasitamab yielded ORR of 57.5% with a CRR of 40%. The median duration of response was 43.9 months. This study included patients with transformed lymphoma, however, the subgroup analysis of response for these patients has not yet been published [26].

Preclinical data have demonstrated that len is a potent enhancer of NK cell-mediated and monocyte-mediated tumor-cell antibody-dependent cellular cytotoxicity. It also enhances interferon-gamma production via Fc-gamma receptor-mediated signaling in response to IgG [28]. This may explain the activity of our regimen, compared to the poor response to single-agent len for patients with RS. Moreover, Nurse-Like Cells, which are similar to M2 macrophages, have potent immunosuppressive functions, facilitating treatment resistance in patients with CLL. In vitro studies have demonstrated that both len and HDMP can mitigate the protective activity of Nurse-Like Cells on leukemia cells. Thus, the synergistic effects of len and HDMP on the tumor micro-environment may help facilitate a better response to immunotherapy [29,30]. The principal effect of len on the tumor micro-environment, as opposed to a direct cytotoxic effect, may explain why responses were seen at a doses of 5 mg, 15 mg and 20 mg.

Len-based regimens have been found to be particularly effective in transformed lymphomas. In a retrospective study of 62 patients with aggressive lymphomas treated with len-based regimens, Rodgers et al. demonstrated that for patients with transformed follicular lymphoma, the ORR was 63%, with a median PFS of 24 months. This was significantly better then de-novo DLBCL, which has an ORR of 43.5% and median PFS of 4.6 months with len-based regimens [31]. This was also found in a subset analysis of the NHL-003 study of len monotherapy where 23 patients with transformed FL were found to have an ORR of 57% with a median PFS of 7.7 months [32]. Len-based regimens may be more efficacious for patients with transformed lymphomas, such as RS, because of the well-established clinical efficacy of len for patients with indolent lymphoma including CLL [33,34]. This may explain why we saw improved clinical activity of our combination. 

Our study has several limitations. First, the results reported are the combined findings of an initial case series and then a phase 1 clinical trial that closed early secondary to slow accrual. Second, we were not able to perform clonality testing between RS and CLL at the time of diagnosis: however, most RS cases (≥80%) are clonally related, and thus it would fair to assume that most of our patients in our study had clonally related RS [9]. Furthermore, RS is typically EBV-negative, and all patients in our study were EBV-negative [9]. Lastly, by Richter’s prognostic score, most patients were either intermediate or high-risk, consistent with a high-risk patient population with a poor prognosis [20] Third, our regimen was designed such that len dosing was escalated during each cycle, with a starting dose of 5 mg. This is because patients with CLL can develop tumor flare, tumor lysis syndrome, as well as worsening myelosuppression when starting at higher doses. However, the typical starting dose of len when treating patients with DLBCL is 25 mg. By starting at a lower dose of len, some patients may progress prior to escalating to a therapeutic dose of len. Nevertheless, responses were seen at doses as low as 5 mg.To improve efficacy, future studies that investigate len for patients with RS could consider starting at a higher starting dose, with the understanding that this may increase toxicity. 

## 5. Conclusions

Richter’s Syndrome (RS) represents a transformation of CLL, typically into diffuse large B-cell lymphoma (DLBCL) or, rarely, into classic Hodgkin lymphoma (cHL) or plasmablastic lymphoma, with an overall poor prognosis. We performed a phase 1 clinical trial of obinutuzumab, HDMP, and len for patients with RS that demonstrated clinical activity without any unexpected safety signals. Three patients were able to undergo either allogeneic or autologous stem cell transplantation and currently remain in remission. Further investigation into len based treatment approaches is warranted for patients with RS. 

## Figures and Tables

**Table 1:** Treatment Protocol.

	Obinutuzumab	HDMP	Lenalidomide
Dose Level -1	1000 mg IV Days 1, 8, & 15	1000 mg/m^2^ IV Days 1–5	2.5 mg Daily
Cycle 1	1000 mg IV Days 1, 8, & 15	1000 mg/m^2^ IV Days 1–5	5 mg Daily
Cycle 2	1000 mg IV D1	1000 mg/m^2^ IV Days 1–5	D1 1 mg daily D15 15 mg daily
Cycle 3	1000 mg IV D1	1000 mg/m^2^ IV Days 1–5	D1 20 mg daily D15 25 mg daily
Cycle 4	1000 mg IV D1	1000 mg/m^2^ IV Days 1–5	25 mg daily
Cycle 5	1000 mg IV D1		25 mg daily
Cycle 6	1000 mg IV D1		25 mg daily
Cycle 7+			25 mg daily

**Table 2:** Patient Characteristics.

	Patient 1	Patient 2	Patient 3	Patient 4	Patient 5	Patient 6	Patient 7
Age	54	71	84	73	64	61	68
Gender	M	F	M	F	M	F	M
ECOG	0	1	1	1	0	0	2
Rai Stage	2	1	1	2	1	2	4
Cytogenetic Abnormalities	Del 11q & del 13q	Del 13q	Trisomy 12; Del 13q	Trisomy 12	Complex including Trisomy 12; del11q; del17p, del13q	Complex including del11q; del13q; and del17p	Complex including del13q and del17p
IGHV Mutational Status	Unmutated	Mutated	Mutated	Mutated	N/A	Unmutated	Mutated
CLL-IPI	4	4	5	4	N/A	10	8
Transformation Histological Subtype	DLBCL	DLBCL	DLBCL	DLBCL	cHL	DLBCL	DLBCL
Time to Transformation	48 months	0 months	423 Months	84 months	66 Months	19 Months	124 Months
# Prior CLL Treatment	2	0	1	0	1	0	7
Previous Treatment with Kinase Inhibitor	yes	No	Yes	No	No	No	Yes
# of Treatment for RS	2	1	0	0	0	0	0
Lugano stage at time of Transformation	IV	III	II	II	III	IV	IV
Bone marrow involvement of RS	Yes	No	No	No	No	No	Yes
Double/Triple HIT	No	No	No	No	No	No	No
Double Expressor	No	No	Yes	No	No	No	No
Richter’s Prognostic Score	Intermediate Risk	Low-Risk	Intermediate-Risk	Low-Risk	High-Risk	Intermediate-Risk	High-Risk

Abbreviations: ECOG = Eastern Cooperative Oncology Group; IGHV = Immunoglobulin Heavy Chain Variable; CLL = Chronic Lymphocytic Leukemia; IPI = International Prognostic Index; RS = Richter’s Syndrome; DLBCL = Diffuse Large B-cell Lymphoma; cHL = Classic Hodgkin’s Lymphoma.

**Table 3:** Disposition of Patients.

Patient Number	Number of Cycles of Treatment	Highest Lenalidomide Dose	Best Response	Proceeded to Autologous/Allogeneic Stem Cell Transplant	Reason for Stopping Treatment
1	2	15 mg	SD	No	Insufficient clinical response
2	2	10 mg	PD	No	PD
3	1	5 mg	SD	No	Insufficient clinical response
4	5	15 mg	PR	Yes	Autologous Stem Cell Transplant
5	5	5 mg	CR	Yes	Allogeneic Stem Cell Transplant
6	4	20 mg	CR	Yes	Allogeneic Stem Cell Transplant
7	2	10 mg	PD	No	PD

**Table 4:** Adverse Events.

	All Grades, %	Grade 1/2, %	Grade 3/4, %
Neutropenia	57	29	29
Thrombocytopenia	43	29	14
Anemia	43	29	14
Insomnia	43	43	0
Fatigue	57	57	0
Dyspnea	14	14	0
Constipation	29	29	0
Headache	29	29	0
Weight Gain	14	14	0
Cramping	29	29	0
Bruising	14	14	0
Right Leg Pain	14	14	0
Weakness	14	14	0
Rash	14	14	0
Weight Loss	43	43	0
Hypokalemia	14	14	0
Mood Swings	14	14	0
Memory Loss	14	14	0
Dry Mouth	14	14	0
Pulmonary Embolism	29	0	29
Sinusitis	14	14	0
Edema	29	29	0
Hypertension	14	14	0
Loss of Bladder Control	14	14	0
Infection	29	14	14
Cough	14	14	0
Thrush	14	14	0
GERD	14	14	0
Diarrhea	14	14	0
Intra-abdominal Hemorrhage	14	0	14

## Data Availability

Not applicable.
